# Clinical relationship between dry eye disease and uveitis: a scoping review

**DOI:** 10.1186/s12348-022-00323-0

**Published:** 2023-01-30

**Authors:** William Rojas-Carabali, Germán Mejía-Salgado, Carlos Cifuentes-González, Valeria Villabona-Martínez, Nicolás Doménico Barraquer-López, David Valdés-Arias, Alejandra de-la-Torre

**Affiliations:** 1 Neuroscience Research Group (NEUROS), Neurovitae Center for Neuroscience, Institute of Translational Medicine (IMT), School of Medicine and Health Sciences, Universidad del Rosario, Bogotá, Colombia; 2 Ophthalmology Interest Group, Neurovitae Center for Neuroscience, Institute of Translational Medicine (IMT), School of Medicine and Health Sciences, Universidad del Rosario, Bogotá, Colombia

**Keywords:** Dry eye disease, Uveitis, Ocular inflammation, Ocular surface, Scoping review

## Abstract

This scoping review examined the relationship between Dry Eye Disease (DED) and Uveitis. We searched Pubmed, Embase, and LILACS databases for articles in which at least one patient had DED and uveitis concomitantly. The search produced 2381 records, and 24 studies were included in the qualitative synthesis. We concluded that DED and uveitis of any etiology could appear concomitantly in patients of any age. However, both diseases seem to coexist more frequently in middle-aged women and cases of anterior uveitis. Therefore, it is crucial that ophthalmologists actively look for the coexistence of ocular surface abnormalities, especially in patients with these characteristics. Future studies should establish and quantify the risk factors and pathophysiological mechanisms of this coexistence to achieve an early diagnosis of both etiologies and comprehensive management of these patients.

**Supplementary Information:**

The online version contains supplementary material available at 10.1186/s12348-022-00323-0.

## Background

Inflammation and autoimmunity are pathological processes in many diseases affecting multiple tissues, and the eye is no exception. Dry eye disease (DED) has been recently considered an ocular surface autoimmune disorder [1], where cornea, conjunctiva, eyelids, lacrimal glands, goblet cells, and meibomian glands can be involved. According to The Dry Eye Workshop II (DEWS II), published by the Tear Film and Ocular Surface Society (TFOS), DED [2] can be caused by insufficient tear production or increased evaporation of the tears due to decreased lipid production from dysfunctional meibomian glands or a combination of both. In all cases, inflammation and neurosensorial disorders play an important role. Patients with DED complain of discomfort, visual disturbance, burning and foreign body sensation, conjunctival hyperemia, and photophobia [3]. DED has a prevalence of 6.8% of the US adult population [4] and can affect any gender and age, but the 40-50 age group is the most affected (5-50%) [5–7], and in some studies, female predominance has been reported [4, 8, 9].

On the other hand, uveitis is the term used to describe the inflammation of the eye’s pigmented and vascularized middle layer. According to the affected anatomical site, uveitis is classified as anterior, intermediate, posterior, and panuveitis [10]. Although numerous cases are idiopathic, some uveitis are related to autoimmune and infectious etiologies [11]. Compared to DED, uveitis is a less common problem, with an incidence of 17 to 52 per 100,000 inhabitants/year and a prevalence of 38 to 714 cases per 100,000 inhabitants. It can be present in any age group, but adults from 20 to 50 years old are the most affected (60-80%) [12]. Uveitis can share symptoms with DED, such as photophobia, blurred vision, and decreased vision; nevertheless, they differ in the pattern of ocular pain, described as neuropathic pain for DED [13] and dull pain around or in the eye, which may worsen when focusing on uveitis. Additionally, the pattern of redness is described as a ciliary injection in uveitis and diffuse hyperemia in DED [14]. Some severe or long-lasting uveitis complications are glaucoma, cataract, cystoid macular edema, chorioretinal neovascularization, epiretinal membranes, and blindness [15].

Experimental studies have shown that DED and uveitis share some pathophysiological aspects (molecular signaling pathways), such as the role of Th1 lymphocytes in diseases initiation, IL 17 and Th17 expression, metalloproteinases elevation and activation, and infiltration of innate immune cells, such as macrophages and dendritic cells [16]. Likewise, the role of NLRP1, NLRC4, AIM2, and NLRP3 inflammasomes have been studied in the pathogenesis of DED, uveitis, and other diseases, with promissory findings until now [17]. Moreover, patients with uveitis have elevated concentrations of inflammatory cytokines and chemokines as IL-1RA and IL23 in tear samples compared to controls without uveitis (*p* < 0.05), and these cytokines’ profile differs according to the anatomical location of uveitis with higher concentrations when inflammation comprises the anterior segment, suggesting that the intraocular inflammation could have a negative impact in the ocular surface [18].

Although studies have shown that DED and uveitis share some pathophysiological mechanisms and suggest that these diseases could coexist, few studies explore the implication this phenomenon could have in clinical practice [18]. Therefore, this scoping review aimed to summarize the current literature regarding the clinical relationship between DED and uveitis.

## Materials and methods

We performed a scoping review of the literature to assess the relationship between DED and uveitis. First, we systematically searched the literature to identify original articles, case reports, case series, cross-sectional, and case-control studies. The search was performed using PubMed database (https://pubmed.ncbi.nlm.nih.gov accessed on 24 of September 2021) with the following MeSH terms ((“Uveitis” [All Fields] OR “Intraocular inflammation” [All Fields]) AND (“Dry eye” [All Fields] OR “Dry eye syndrome” [All Fields]’)); Embase database (https://www.embase.com) where Entree terms were adapted to ((“Uveitis” [All FIelds] OR “Intraocular inflammation” [All Fields]) AND (“Dry eye” [All Fields] OR “Dry eye syndrome” [All Fields]’)), and LILACS (https://lilacs.bvsalud.org/es/) using the following search ((“Uveítis” [words] OR “inflamación intraocular” [words]) AND (“Síndrome de Ojo Seco” [words] OR “Ojo Seco” [words]). After the exclusion of duplicated records, three pairs of review authors (WRC, GMS, CCG, VVM, NDB, and DV) independently examined the titles and abstracts identified by the electronic searches and decided if the record would be included or not. If there were discrepancies, a third reviewer made the decision (ADLT). Then, the same reviewers’ groups read the full text and included the articles in which at least one patient had reported DED and uveitis concomitantly. We excluded narrative reviews and articles related to animal evidence exclusively. Due to the diversity in the terms related to dry eye (keratoconjunctivitis sicca (KCS), dry eye, dry eye syndrome, DED, and dry eye symptoms) and considering the long period of observation of studies (1975 - 2021), we decided to homogenize using the term DED if any of the terms were reported in the studies [19, 20].

**Fig. 1:**
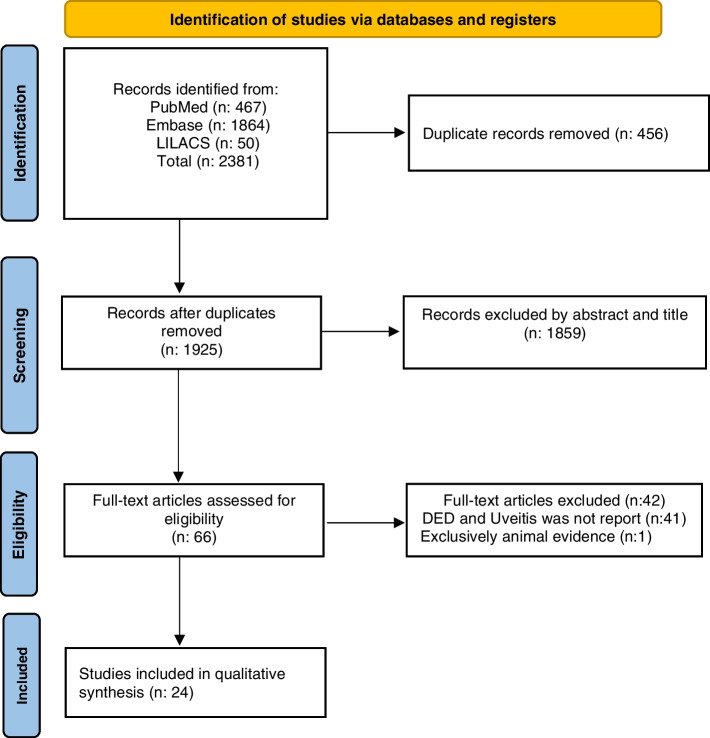
Prisma Flow Diagram

We extracted the following data from the included articles: type of study, number of patients included in the study, number of patients with uveitis, number of patients with uveitis and DED, mean age of uveitis presentation, gender, anatomical localization, etiology, ocular examinations findings, and DED test. Finally, we performed a narrative synthesis of the studies found (Fig. 1).

## Results

The initial search retrieved a total of 2381 articles. After removing duplicates, 1925 articles were reviewed for title and abstract, obtaining 66 articles for full-text review. After that, we obtained 24 studies that were included in the qualitative synthesis. We divided these articles according to the uveitis etiology reported as non-infectious (Table 1) and infectious (Table 2). Then, we grouped the articles by type and summarized them. More detailed information about the articles selected is available in the Supplementary material.

**Table 1:** Principal findings of articles regarding non-infectious uveitis and DED

Author, year	Title	Design of study	N total N uveitis Nuveitis and DED	Gender of patients with uveitis	Localization of uveitis	Etiology	Mean age of uveitis presentation (years)	DED tests	Relationship between diseases
Rosenbaum JT et al. [21], 1987	Chronic Anterior and Posterior Uveitis and Primary Sjogren’s Syndrome	Case series	8 8 8	Male:1 Female: 7	Panuveitis: 8	Sjogren’s syndrome 8/8	54.1	8/8 patients with uveitis had a low Schirmer test (≤ 9 mm)	Description of patients with Sjogren’s syndrome. Among patients with uveitis 100% (8/8) had dry eye
Bridges AJ et al. [22], 1992	Acute iritis associated with primary Sjögren’s syndrome and high-titer anti-SS-A/Ro and anti-SS-B/La antibodies. Treatment with combination of immunosuppressive therapies.	Case report	1 1 1	Male: 0 Female: 1	Anterior: 1	Primary Sjögren’s syndrome 1/1	52	The patient had KCS confirmed by Schirmer test and rose-Bengal stain	Description of a 52 year old patient with Sjogren’s syndrome who developed uveitis and KCS
Hegab SM et al. [23], 1997	Ocular sarcoidosis in Kuwait with a review of literature	Case series	18 18 6	Male: 8 Female: 10	- Anterior: 5 - Intermediate + Anterior: 10 - Panuveitis: 3	Ocular sarcoidosis 18/18	19	1/6 had severe dry eyes noted by Schirmer’s test reading 0	Description of patients with ocular sarcoidosis. Among patients with uveitis 33.3% (6/18) had dry eye
Araki T et al. [24], 2001	Two elderly patients with sarcoidosis and Sjögren’s syndrome	Case series	2 2 2	Male: 0 Female: 2	N/A	Sjögren’s syndrome and Sarcoidosis 2/2	70	2/2 patients with uveitis had a positive Schirmer test and rose Bengal staining	Description of 2 elderly patients with sarcoidosis and Sjögren’s syndrome who developed dry eye and uveitis
Zagórski Z et al. [25], 2001	[Zinsser-Engman-Cole syndrome (dyskeratosis congenita) with severe sicca syndrome, panuveitis and corneal perforation--a case report].	Case report	1 1 1	Male: 1 Female: 0	Panuveitis: 1	Zinsser-Engman-Cole syndrome 1/1	46	The patient with uveitis had a Schirmer test I with OD 0 mm and OS 9 mm	Description of a 46 year old patient with Zinsser-Engman-Cole syndrome who developed uveitis and dry eye
Ramos-Casals M et al. [26], 2004	Sarcoidosis or Sjögren Syndrome? Clues to Defining Mimicry or Coexistence in 59 Cases	Case series	5 2 2	Male: 0 Female: 2	Anterior: 2	Sjögren’s syndrome and Sarcoidosis 5/5	50.5	1/2 of the patient with uveitis had non-specified positive ocular tests 1/2 of the patient with uveitis had positive Schirmer test and Rose Bengal staining	Description of patients with Sarcoidosis and Sjogren’s syndrome. Among patients with uveitis 100% (2/2) had abnormalities in ocular surface test suggestive of dry eye
Lendechy-Velázquez M, et al. [27], 2019	IgG4-related disease	Case Series	2 1 1	Male: 1 Female:0	Anterior: 1	IgG4 related disease 2/2	67	The patient with uveitis had AO with positive Schirmer test	Description of a 67-year-old patient with IgG4-related disease who developed uveitis and dry eye
Cui D et al. [28], 2020	Concurrent primary Sjögren’s syndrome and isolated ocular sarcoidosis presenting with bilateral corneal scarring and dry eye	Case report	1 1 1	Male:1 Female:0	Anterior: 1	Sjogren’s syndrome and sarcoidosis 1/1	60	The patient had significant bilateral bulbar conjunctival lissamine green staining and corneal punctate epithelial erosions	Description of a 60-year patient with Sjogren’s syndrome and sarcoidosis who developed uveitis and dry eye
Babu K et al. [29], 2021	Clinical Profile in Genetically Proven Blau Syndrome: A Case Series from South India	Case series	7 5 4	Male: 2 Female: 3	Anterior: 2 Panuveitis: 3	Blau syndrome 7/7	10	2/5 patients with uveitis had a low Schirmer test (≤ 6 mm)	Description of patients with Blau syndrome. Among patients with uveitis 80% (4/5) had dry eyes.
Chylack LT et al. [30], 1975	Ocular manifestations of juvenile rheumatoid arthritis	Cross sectional study	210 36 3	Male: 6 Female: 30	N/A	Juvenile Rheumatoid Arthritis 210/210	4.3 ± 1	3/36 patients with uveitis had positive Schirmer test	Description of patients with juvenile rheumatoid arthritis. Among patients with uveitis 8.3% (3/36) had KCS
Kanski JJ et al. [31], 1988	Uveitis in Juvenile Chronic Arthritis: Incidence, Clinical Features and Prognosis	Cross sectional study	315 315 5	N/A	Anterior: 315	Juvenile Chronic Arthritis 315/315	6	5/315 patients with uveitis had rose Bengal typical staining pattern for KCS	Description of patients with JCA. Among patients with uveitis 1.58% (5/315) had KCS
Mastropasqua L et al. [32], 1996	Tear deficiency in Fuchs’ intermediate uveitis	Cross sectional study	30 30 15	N/A	Intermediate 30/30	Fuchs’ (heterochromic) uveitis 30/30	N/A	15/30 patients with uveitis had abnormal Schirmer’s test I, tear film breakup time and Ferning test	Description of patients with Fuchs’ heterochromic uveitis. Among patients with uveitis 50% (15/30) had tear deficiency abnormalities
Evans M et al. [33], 2007	Differences in clinical findings between Caucasians and African Americans with biopsy-proven sarcoidosis.	Cross sectional study	81 33 1	Male: 9 Female: 24	- Anterior: 16 - Anterior + Intermediate: 1 - Intermediate: 3 - Posterior: 11 - Panuveitis: 2	Sarcoidosis 81/81	N/A	N/A	Description of patients with sarcoidosis. Among patients with uveitis 3% (1/33) had KCS
Ungprasert P et al. [34], 2016	Clinical characteristics of ocular sarcoidosis: A population-based study 1976-2013	Cross sectional study	345 14 1	Male: 3 Female: 11	- Anterior: 9 - Anterior and intermediate: 1 - Intermediate: 2 - Posterior: 1 - Panuveitis: 1	Sarcoidosis 345/345	52.5	N/A	Description of a 50-year-old patient with sarcoidosis who developed uveitis and dry eye
Caimmi C et al. [35], 2018	Clinical correlates, outcomes, and predictors of inflammatory ocular disease associated with rheumatoid arthritis in the biologic era	Cross sectional study	92 14 1	Male: 5 Female: 9	Anterior: 14	Rheumatoid arthritis 92/92	N/A	A diagnosis of dry eye was made when at least 1 of the followings tests were positive: rose Bengal/ Lissamine green corneal surface staining or Schirmer test < 5 mm	Description of patients with rheumatoid arthritis. Among patients with uveitis 7.1% (1/14) had dry eye
Maryam T et al. [36], 2019	Ocular surface disease in patients with panuveitis: Incidence and characteristics	Cross sectional study	101	Male: 33 Female: 68	Panuveitis: 101	Idiopathic uveitis 12/101 Sarcoidosis 11/101 Non-specified 78/101	50.1	N/A	Description of patients with panuveitis sarcoidosis, idiopathic, and other non-specified etiologies. Among patients with uveitis 8.9% (9/101) had dry eye signs
			101						
			9						
Degirmenci C et al. [37], 2020	Evaluation of ocular surface and meibomian glands in patients with uveitis related to oligoarticular juvenile idiopathic arthritis	Cross sectional study	17 17 2	Male: 5 Female: 12	N/A	Juvenile Idiopathic Arthritis 17/17	17 ± 5.69	2/17 patients with uveitis had an abnormal Schirmer 1 test (≤ 5 mm)	Patients with oligoarticular JIA who developed uveitis and dry eye with a significant higher total meiboscores compared to controls, which indicates a possible evaporative dry eye tendency in this entity
Pivetti Pezzi P et al. [38], 2004	Vogt-Koyanagi-Harada Syndrome and Keratoconjunctivitis Sicca	Case Controls	32 16 > 16	Male: 8 Female: 24	NA	Vogt-Koyanagi-Harada 16/32 Unspecified bilateral diffuse uveitis 16/32	41 ± 13.40	From the VKH group: 16/16 had a BUT < 5 seconds, 12/16 had a Schirmer test ≤3 mm and an abnormal Ferning Test (type III–IV), 10/16 had an abnormal corneal fluorescein and/or rose Bengal staining (Van Bijsterveld score ≥ 4) From the control group Unspecified uveitis group: 5/16 had a BUT < 5 seconds and an abnormal Ferning Test (type III–IV), 2/16 had a Schirmer test ≤3 mm and an abnormal corneal fluorescein and/or rose Bengal staining (Van Bijsterveld score ≥ 4)	Description of patients with VKH syndrome. Among patients with uveitis 100% (16/16) had abnormalities in ocular surface suggestive of KCS Patients with VKH syndrome had a higher incidence of KCS than control patients who had others bilateral diffuse uveitis
Akinci A et al. [39], 2007	Keratoconjunctivitis Sicca in Juvenile Rheumatoid Arthritis	Case Controls	128 15 2	N/A	N/A	Juvenile Rheumatoid Arthritis 64/64	N/A	2/15 patients with previous uveitis had KCS. A diagnosis of KCS was made when 2 out of 4 of the followings test were positive: Schirmer test ≤5 mm, BUT ≤10 seconds, rose Bengal staining score > 3 points or fluorescein staining score ≥ 1	Patients with juvenile rheumatoid arthritis. Among patients with uveitis 13.3% (2/15) had KCS There were no significant differences in TBUT and Schirmer test results between children with or without a history of uveitis in patients with JRA
Abbouda A et al. [40], 2017	Psoriasis and Psoriatic Arthritis-Related Uveitis: Different Ophthalmological Manifestations and Ocular Inflammation Features	Case Controls	117 117 21	Male: 59 Female: 58	- Anterior: 99 - Intermediate: 3 - Posterior:10 - Panuveitis: 5	Psoriasis 92/117 Psoriatic arthritis 25/117	52.5 ± 16.0	N/A	Dry eye was the 5th most common complication 13.3% (21/117) after vitritis 41.1% (65/117), cataract 29.7% (47/117), posterior vitreous detachment 25.9% (41/117) and elevated intraocular pressure 17% (27/117)

**Table 2:** Principal findings of articles regarding infectious uveitis and DED

Author	Title	Design of study	N total N uveitis N uveitis and DED	Gender	Localization of uveitis	Etiology	Mean age of uveitis presentation (years)	DED test	Relationship between diseases
Merle H et al. [41], 2002	Ocular lesions in 200 patients infected by the human T-cell lymphotropic virus type 1 in Martinique (French West Indies)	Case series	200 29 11	Male: 11 Female:18	- Panuveitis 10/29. - Intermediate and posterior 4/29. - Intermediate 5/29. - Anterior and intermediate 7/29 - Anterior 3/29.	Human lymphocytic virus - Type 1200/200	55.6	11/29 patients had KCS. A diagnosis of KCS was made when 2 out of 3 of the following tests were positive: Schirmer test 1 < 10 mm, BUT < 10 seconds, rose bengal staining score > 3 points (Van-Bijsterveld score)	Uveitis group had a slightly higher prevalence of KCS 37.9% (11/29) vs non-Uveitis group 36.8% (63/171)
Öztürk C et al. [42], 2021	Bilateral Acute Anterior Uveitis and Corneal Punctate Epitheliopathy in Children Diagnosed with Multisystem Inflammatory Syndrome Secondary to COVID-19	Case series	5 5 3	Male: 1 Female: 4	Anterior 5/5	SARS COV 2 5/5	9.4	3/5 patients with uveitis had an abnormal Schirmer test (≤ 7 mm) and corneal fluorescein staining (≥ 2 on the modified Oxford scale)	Description of 5 pediatric patients with uveitis secondary to COVID 19 infection, 60% (3/5) with abnormalities in ocular surface and tear function test
Hoeksema L et al. [43], 2017	Risk Factors for Secondary Glaucoma in Herpetic Anterior Uveitis	Cross-sectional study	73 73 25	Male: 45 Female: 28	Anterior 73/73	- Herpes simplex virus 54/73 - Varicela zoster virus 19/73	50	N/A	Dry eye was the third most common complication 34% (25/73) after Keratitis 59% (43/73) and Elevated IOP 75% (55/73) in uveitis secondary to herpes infection
Babu K et al. [44], 2020	Diagnostic Markers in Ocular Sarcoidosis in A High TB Endemic Population–A Multicentre Study	Case–control study	368 368 43	Male: 193 Female: 175	- Panuveitis 112/368 - Posterior 138/368 - Intermediate 53/368 - Anterior 58/368 - Others 7/368	- Sarcoidosis 61/368 (Group A - GA) - Tuberculosis 307/368 (Group B - GB)	GA: 43 GB: 38	36/42 patients from sarcoidosis group had a low Schirmer test (≤ 10 mm) 7/42 patients from the tuberculosis group had a low Schirmer test (≤ 10 mm)	Schirmer tests were significantly lower in patients with Sarcoidosis 85.7% (36/42) than in Ocular tuberculosis 16.6% (7/42) which could help differentiate ocular sarcoidosis in a high TB endemic population (OR:30, CI 95%)

### Non-infectious etiology

We found six case series and three case reports between 1987 and 2021 describing an association between DED and autoimmune uveitis. One case report and one case series described patients with Sjögren Syndrome (SS) that developed anterior and/or posterior uveitis [21, 22]. Four studies describe a relationship between ocular sarcoidosis (OcSar) and SS [23, 24, 26, 28]. The other three describe cases of IgG4-related disease [27], Zinsser-Egman-Cole syndrome [25], and Blau syndrome [45], in which patients presented uveitis and clinical features of DED. Interestingly, most patients were women with anterior uveitis. The age range of uveitis presentation was 10 to 70 years old.

Additionally, there were eight cross-sectional studies (one analytic, seven descriptive), uveitis was more common in females, and the mean age of presentation ranged from 3.3 to 52.5 years old. Anterior uveitis was the most frequent anatomic localization, even though some studies involved cohorts of patients with only one type of uveitis (anterior, intermediate, or panuveitis). The most commonly related autoimmune disease was Juvenile idiopathic/rheumatoid arthritis (JIA) (25 patients) [30, 31, 37], followed by Fuchs’ heterochromic uveitis (FHU) (15 patients) [32], undetermined (9 patients) [36], Sarcoidosis (2 patients) [33, 34] and Rheumatoid arthritis (RA) (1 patient) [35].

We found three case-control studies that evaluated the ophthalmological manifestations in patients with JIA [39], Psoriatic Arthritis-Related Uveitis (PsA-related uveitis) [40], and Vogt-Koyanagi-Harada syndrome (VKH) [38], the mean age of uveitis presentation ranged from 41 to 52.5 years, with a proportional sex ratio in JIA but with female predominance in VKH. Anterior uveitis was the most common localization, followed by posterior, panuveitis, and intermediate. Interestingly, DED was present in 13.3% of patients with JIA (2/15) [39] and 17.9% PsA-related uveitis (21/117) [40], and 100% of VKH cases (16/16) had abnormalities in ocular surface suggestive of KCS [38].

Rosenbaum et al. [21] described eight patients with SS and uveitis with a Schirmer test positive below 10 mm; all of them were women between 29 and 55 years. Likewise, Ramos Casals et al. [23] presented a case series of patients with Sarcoidosis and SS who all had sicca syndrome (5 with xerophthalmia, 4 with xerostomia). Among them, 2 patients had anterior uveitis with abnormalities on the Schirmer test suggestive of DED. Tahvildari et al. [36] characterized the corneal, conjunctival, and eyelid margin abnormalities in patients with panuveitis and found a prevalence of 44.5% of ocular surface abnormalities and 48.5% had Meibomian gland dysfunction. Interestingly, 20% had dry eye signs, and the most common cause of uveitis in patients with ocular surface disease was idiopathic (26.8%) and sarcoidosis (24.4%), concluding a higher incidence of ocular surface, corneal, and eyelid margin disease in patients with panuveitis. Similar findings were described by Aoki et al. [46], who compared the tear function in OcSar, VKH, and healthy subjects to elucidate the association between OcSar and DED. The Schirmer 1 Test values were significantly lower in the OcSar patients than in the VKH patients (*P* = 0.004) and control subjects (*P* = 0.001). They conclude that the neural reflex arc and lacrimal gland system, which attenuate the vicious cycle between the tear film and ocular surface epithelium in DED, are significantly impaired in OcSar cases, indicating a possible association between OcSar and DED.

Degirmenci et al. [37] performed a case-control study in patients with JIA and chronic bilateral uveitis compared with controls, evaluating the presence of DED and Meibomian gland dysfunction. They found no significant differences between groups regarding age, mean intraocular pressure, mean Schirmer 1 test value, tear film breakup time (TBUT), and Oxford staining score. However, they found that patients with oligoarticular JIA had higher meiboscores than normal subjects, which indicates a possible evaporative dry eye tendency in these patients. Similarly, Akinci et al. [39] studied patients with JIA, of which 23.4% of patients had uveitis, and did not find a significant difference in the Schirmer test and TBUT in this group of patients.

Other less common autoimmune diseases, such as FHU, VKH, and Behçet, have also been related to tear deficiency. Mastropasqua et al. [32] found that 15 out of 30 patients with FHU had tear deficiency noted by abnormal Schirmer 1 test, tear film BUT, and Ferning’s test. There was a significant difference in the test results between the affected eyes and the fellow unaffected eyes (*P* < 0.001). Piveti et al. [38] performed tear function tests (BUT, Schirmer test, fluorescein, and rose bengal staining) in 16 VKH patients compared with 16 control with diffuse uveitis. They found that patients with VKH syndrome had a higher incidence of DED when compared to controls. Karaca et al. [47] evaluated the ocular surface and meibography of 25 right eyes of patients with inactive Behçet’s uveitis (Group 1), and 25 right eyes of 25 healthy individuals (Group 2). They did a Schirmer 1 test, tear film BUT, ocular surface staining with fluorescein and Oxford scoring, and ocular surface disease index (OSDI) score assessment. Also, lower and upper eyelid Meibomian glands were examined with the infrared filter of the slit-lamp biomicroscope. Schirmer test and film breakup time were significantly lower in group 1 in comparison with group 2. Oxford scale and OSDI scores were higher in group 1. There was no significant difference in the upper and lower meiboscores. They conclude that despite the tendency toward DED, Behçet’s uveitis is not associated with quantitative meibomian gland changes, which is demonstrated by gland dropout with meibography.

### Infectious and other etiologies

Regarding infectious etiologies, we found two case series and one cross-sectional study. All uveitis were viral, caused by Human T-cell Lymphotropic Virus Type 1 (HTLV-1) [41], Herpes Virus [42], and SARS-CoV-2 [43]. The mean age of uveitis ranges between 9.4 and 55.6 years old, anterior uveitis was the most common localization of inflammation, followed by panuveitis, and women were the most affected. In a cross-sectional study, Kalpana et al. [29] compared ocular characteristics between uveitis secondary to sarcoidosis and tuberculosis, finding that patients with a low Schirmer test had a higher risk of OcSar.

Merle et al. [41] studied 200 patients infected by HTLV-1, 77 (38.5%) were seropositive, and 123 (61.5%) had HTLV-1-associated myelopathy/tropical spastic paraparesis (HAM/ TSP). Uveitis was found in 29 cases (14.5%). For diagnosing DED, at least 2 out of 3 tests of the followings had to be positive: Schirmer 1 test < 10 mm; BUT < 10 seconds, pink bengal test > 3 points. DED existed in 74 patients (37%). In patients with HAM/ TSP, uveitis was more frequent among younger patients, patients with earlier onset of HAM/TSP, and patients with severe motor disabilities. The sicca syndrome related to HTLV-1 virus differs from primary or secondary Sjögren syndrome because it does not reveal any of the immunologic anomalies generally seen in this disease. Finally, Ozturk et al. [42] described the case of 5 pediatric patients with bilateral non-granulomatous acute anterior uveitis secondary to the multisystem inflammatory syndrome in children (MIS-C) due to COVID-19 infection, and of those, 60% (3/5) with abnormalities in ocular surface and tear function test.

## Discussion

Understanding the pathophysiological mechanisms of diseases is a primary requirement when proposing diagnostic and therapeutic strategies [16]. In the case of eye diseases, many share clinical signs and symptoms that make their approach complex and even more when two conditions are found in the same patient. A clear example is a relationship between glaucoma and DED, diseases known to coexist, mainly because glaucoma medications are a risk factor for developing DED [48]. However, the relationship between DED and uveitis has been poorly explored, a relationship that has a pathophysiological theoretical basis, considering that both are diseases in which the immune system plays an important role [16].

### Molecular relationship between both entities: a hypothesis

Even though DED and uveitis are not specific nosological entities alone, both entities share an inflammatory pathway by definition and can coexist. Based on the current literature, we draw some hypotheses and inferences in the pathophysiology that can underlie in patients with uveitis and DED overlapping. It has been demonstrated that HLA-DR expression by conjunctival cells is increased in patients with uveitis and DED compared with those with vernal keratoconjunctivitis and normal subjects [49]. Likewise, it is known that high HLA-DR expression in conjunctival epithelial cells associated with high conjunctival staining may identify a subgroup of DED patients prone to epithelial disease that possibly need a different approach from current treatment standards [50]. These results suggest an involvement of the Th2 system on the ocular surface in uveitis; thus, exploring the ocular surface in uveitis may represent a new way to understand better the immune pathways involved in this complex disease [49].

Other studies have evidenced that uveitis can modify the cytokine and chemokine profile in aqueous humor and tears. Patients with uveitis have higher tear levels of IL-1β [18], a well-known immune marker of DED [51]. Likewise, these patients have higher tear levels of IL-23 compared with controls [18]. This interleukin plays an essential role in the long-lived memory of T-helper 17 (Th17), which actively mediates chronic inflammation in autoimmune disorders, including DED [52, 53].

Another crucial cytokine in the inflammatory mechanism of diseases is IFN-γ. Hill et al. [54] investigated differences in the T cells in aqueous humor between several types of uveitis and correlated it with clinical phenotype. However, they just found increased percentages of IL-10^+^ −, but not interferon IFN-γ + T lymphocytes in aqueous humor compared with peripheral blood in patients with acute anterior uveitis (AAU), FHU, or chronic panuveitis. As the authors mention, “this could be due to the fact that some patients had a higher baseline expression of IFN-γ + T cells due to their disease being active and stimulation with phorbol myristate acetate failing to augment fully or any further the number of cytokine-positive T cells.” In the same way, Carreño et al. did not find significant differences in tear levels of IFN-γ between uveitis patients and healthy controls [18]. However, they found an increase in the IP-10/CXCL10; this chemokine is often released in the context of inflammation by many cells, including leukocytes, neutrophils, eosinophils, monocytes, and stromal cells, in response to IFN-γ [55].

Additionally, there was a trend towards elevated levels of IL10^+^ T cells in aqueous humor from patients with FHU compared with those from acute uveitis and panuveitis patients. Increased levels of IL10^+^ T cells in aqueous humor compared with peripheral blood were also found in samples from patients with isolated uveitis but not those with associated systemic disease [54]. Interestingly, IL-10 has been related to the disease onset and activity in SS [56–58], a disease that can present both DED and uveitis.

Another relevant cytokine in the pathophysiology of both entities is IL6, related to the intraocular immune response in several types of uveitis and involved in DED pathology, even correlated with eye pain in the latter [59]. Also, it has been evidenced that epithelial cells produce and release chemokines, tumor necrosis factor-alpha (TNF-α), IL-1, IL-6, and IL-8, which amplify the immune response and attract inflammatory cells in DED [60]. Undoubtedly, the action of a single cytokine or chemokine cannot wholly explain the pathophysiology of the uveitis/DED overlapping, and more complex molecular regulators such as inflammosomes may play a fundamental role, as they have emerging importance in regulating ocular surface and anterior segment health and disease [17].

### Non-infectious etiology

The multiple published case series and case reports make us think that coexisting DED and uveitis could be more common than we suppose. This is backed by the cross-sectional studies appraised that showed a relationship between DED and uveitis, especially with anterior chamber compromise, considering that the most common anatomic localization of uveitis in patients who concomitantly had DED was anterior, followed by panuveitis. Tahvildari et al. reported DED in 8.9% (9/101) of patients with panuveitis [36]. Likewise, Caimmi et al. described a cohort of 92 RA patients with ocular inflammatory disease, of which 14 patients had anterior uveitis and one patient had DED concomitantly, with a Schirmer test < 5 mm in 5 minutes and positive staining [35]. These results coincide with previous studies where higher levels of inflammatory markers have been found in patients with anterior and panuveitis compared with intermediate and posterior uveitis [18]. This suggest that inflammation of the anterior chamber might spread locally to the ocular surface, supporting that intraocular inflammation could generate corneal and conjunctival changes.

Regardless of localization or etiology, women were the most frequently affected by concomitant DED and uveitis. This is expected, considering that the Pacific Ocular Inflammation Study reported that although there are no significant differences in incidence rates of uveitis between genders, there is a higher prevalence in females [61], and multiple studies have evidenced that DED is most common in women. Additionally, it is crucial to consider that gender is a common risk factor for autoimmune diseases, such as in JIA, the most frequent systemic disease found in the cross-sectional studies evaluated in the present review [62].

Regarding tear function tests, 50% (15/30) of patients with FHU had impaired tear production, evidenced by the Schirmer test [32]. Moreover, JIA-related uveitis patients had a higher median meiboscore (*p* = 0.041), abnormal Schirmer test results (< 5 mm in 5 min) [30, 35], and ocular surface staining [31] compared to healthy subjects. Thus, patients with uveitis can present both evaporative or aqueous deficiency DED. However, there is a lack of information regarding the risk factors for presenting either.

Case-control studies identified similar information to the previously described, where most patients were women with anterior uveal compromise. For example, a study comparing patients with VKH versus other types of uveitis found that all VKH patients had altered tear function tests, with a statistically significant difference in DED proportion between both groups [38]. However, in another cohort of children with JIA, there were no significant differences in TBUT and Schirmer test results between those with or without a history of uveitis [39]. Therefore, the current literature is controversial, and further research is needed with prospective studies to characterize the risk factors associated with DED in patients with specific etiologies of uveitis.

### Infectious etiology and others

The observational studies that assessed the relationship between DED and infectious uveitis found similar associations. Merle et al. described a cohort of 200 patients infected by the Human T-cell Lymphotropic Virus Type 1 (HTLV-1), where a slightly higher prevalence of DED was observed in the uveitis group (11/29, 37.9%) vs. non-uveitis group (63/171, 36.8%). There was a female: male ratio of 7:4. Four patients had panuveitis, of which three were female. Five had anterior and intermediate uveitis, of which three were female. And two had intermediate uveitis, with a 1:1 relationship [41]. Similarly, in herpetic uveitis, DED was described as the third most common complication (25/73, 34%) after keratitis (43/73, 59%) and elevated IOP (55/73, 75%) [43]. Additionally, abnormalities in ocular surface and tear function tests in pediatric patients were observed in 60% (3/5) of cases with anterior uveitis due to MIS-C secondary to COVID-19. Three patients were female and presented with corneal punctate epitheliopathy [42]. These results suggest a relation between viral infectious uveitis and DED. Infectious mechanisms seem to be involved in dysfunction in tear production or preservation. Additionally, we found an interesting multicenter study performed by Babu et al. that aimed to look at clinical and radiological markers to differentiate OcSar from ocular tuberculosis. They described significantly lower Schirmer test results in patients with sarcoidosis (36/42, 85.7%) than in ocular tuberculosis (7/42, 16.6%), proposing it as a marker that could help differentiate OcSar in a high TB endemic population. Patients with uveitis and low Schirmer test had higher odds of OcSar than TB (OR- 30, CI-95% 9.168 - 98.173) [44].

In general, most studies only describe the frequency of coexistence of both entities, which can vary between 1.58% and 100% depending on the characteristics of the cohort studied. Only one study compared patients with and without uveitis, finding DED more common in patients with uveitis [41]. Another study in patients with Inactive Behçet’s uveitis found a “trend” towards DED as these patients had lower levels of Schirmer 1 test (18.68 vs. 23.69, *p* = 0.017) and mean tear film BUT (10.76 vs. 13.36, *p* = 0.026) compared to healthy controls [47].

Furthermore, it is essential to consider that uveitis is not always an organ-specific condition. In fact, non-infectious uveitis is associated with a systemic autoimmune disease in up to 33% of cases [63]. And it has been shown that these diseases can also be related to alterations of the ocular surface and DED in up to 53% of cases [64], regardless of the presence or not of uveitis. Likewise, it is essential to consider the role that treatment may have since, generally, uveitis and its complications are treated with topical and systemic medications that cause DED, such as corticosteroids, anti-glaucoma agents, cycloplegics, and NSAIDs [65, 66].

This scoping review summarizes the literature regarding the clinical relationship between DED and uveitis; however, many questions remain open. For example, most studies are cross-sectional, so the question of which came first: the DED or the uveitis? Is still unanswered. This is the first step in understanding the coexistence of both diseases. It encourages researchers to continue studying this phenomenon to understand the link between them, their clinical implications, and the need for effective diagnostic mechanisms and treatment approaches in cases where both diseases present concomitantly.

## Conclusion

Observational studies showed that uveitis and DED could appear concomitantly in patients of any age and with any uveitis etiology. However, it seems that middle-aged women are the ones in whom the two diseases coexist more frequently. This could represent clinical evidence of the common pathophysiological pathways found between these inflammatory diseases or maybe just a coincidence because both disorders are more common in adult women. Anyway, it is essential that ophthalmologists actively look for the coexistence of DED, both aqueous and lipo deficient, in patients with uveitis, especially if anterior.

Further research about these disorders’ risk factors and pathophysiological mechanisms is essential, considering that several studies have shown that intraocular inflammation might expand to the ocular surface, evidenced by the increase in cytokines in the tears of patients with uveitis. Therefore, longitudinal studies will allow us to know which of the two diseases appears first and the clinical implications of this association. By increasing our knowledge of this coexistence phenomenon, we could develop diagnostic methods that allow timely diagnosis and therapeutic strategies that will enable the comprehensive management of these patients.

## Supplementary Information

**Additional file 1.**

## Data Availability

All data generated or analyzed during this study are included in Table 1 and Table 2 and Supplementary files.

## Abbreviations

ADLT: Alejandra de-la-Torre
AIM2: Interferon-inducible protein absent in melanoma 2
CCG: Carlos Cifuentes González
COVID-19: Coronavirus disease 2019
DED: Dry eye disease
DEWS II: The Dry Eye Workshop II
DVA: David Valdes-Arias
FHU: Fuchs’ Heterochromic Uveitis
GMS: Germán Mejía Salgado
HAM/ TSP: HTLV-1 associated myelopathy/tropical spastic paraparesis
HLA-DR: Major histocompatibility complex (MHC) II cell surface receptor
HTLV-1: Human T-cell Lymphotropic Virus
HTLV-1: Human T-cell Lymphotropic Virus Type 1
IFN-γ: Interferon gamma
IL: Interleukin
IL-1RA: Interleukin-1 receptor antagonist
IP-10/CXCL10: Interferon gamma-inducible protein 10/ C-X-C motif chemokine 10
JIA: Juvenile idiopathic arthritis
KCS: Keratoconjunctivitis sicca
LILACS: Literatura Latinoamericana y del Caribe en Ciencias de la Salud
MIS-C: Multisystem inflammatory syndrome in children
NDB: Nicolás Domenico Barraquer
NLRC4: Nucleotide-binding domain-(NOD) like receptor family CARD domain-containing protein 4
NLRP1: Nucleotide-binding domain-(NOD) like receptor family pyrin domain containing 1
NLRP3: Nucleotide-binding domain-(NOD) like receptor family pyrin domain containing 3
NSAIDs: Non-steroidal Anti-inflammatory Drugs
OcSar: Ocular sarcoidosis
OR: Odds ratio
OSDI: Ocular surface disease index
PsA-related uveitis: Psoriatic Arthritis-Related Uveitis
RA: Rheumatoid arthritis
SARS-CoV-2: Severe acute respiratory syndrome coronavirus 2
SS: Sjögren Syndrome
TB: Tuberculosis
TBUT: Tear film breakup time
TFOS: Tear Film and Ocular Surface Society
Th17: T-helper 17
TNF-α: Tumor necrosis factor-alpha
US: United States
VEGF: Vascular-Endothelial Growth Factor
VKH: Vogt-Koyanagi-Harada syndrome
VVM: Valeria Villabona Martínez
WRC: William Rojas-Carabali
